# Differential clinical outcomes in ACS: the amplified risk of diabetes in patients treated with drug-coated balloon angioplasty

**DOI:** 10.1186/s43044-025-00686-4

**Published:** 2025-09-12

**Authors:** Sheeren Khaled, Saleh Khouj, Mamdouh Ismail, Ahmed Ebrahim, Ghada Shalaby, Anas Sheikh, Abdullatif Ujami, Mohamed Elsheikh, Mohammed Sadhiq, Ahmed Darwish

**Affiliations:** 1 King Abdullah Medical City, Makkah al Mukarramah, Saudi Arabia; 2 Benha University, Banhā, Egypt; 3 Zagazig University, Zagazig, Egypt

**Keywords:** Acute coronary syndromes, Diabetes mellitus, Type 2, Percutaneous coronary intervention, Treatment outcome

## Abstract

**Background:**

Emerging evidence supports drug-coated balloons (DCBs) as a compelling alternative to stent-based interventions in acute coronary syndrome (ACS), particularly in diabetic populations where lesion complexity and metabolic dysfunction often undermine long-term outcomes. This study explores real-world safety and efficacy of DCB-only angioplasty in ACS patients, with focused analysis of diabetic subgroups.

**Methods:**

A single-center, retrospective cross-sectional analysis was conducted at King Abdullah Medical City between 2019 and 2023. The study enrolled patients presenting with ACS who underwent DCB treatment during this period.

**Results:**

Of 212 patients, 55% had diabetes mellitus. Diabetic individuals exhibited significantly higher comorbid burden—hypertension, dyslipidemia, and prior coronary interventions. Non-ST-elevation myocardial infarction (NSTEMI) predominated among diabetics, and in-stent restenosis (ISR) was more frequently observed than de novo lesions. At one-month follow-up, diabetic patients experienced marginally fewer major adverse cardiovascular events (MACE) than non-diabetics; however, parity was observed at one year. Within the diabetic group, insulin therapy and suboptimal glycemic control (HbA1c > 8.0%) were strongly associated with increased 12-month cardiovascular risk, particularly due to repeat revascularization. Importantly, neither clinical presentation nor angiographic features predicted these risks. Instead, elevated HbA1c and reduced left ventricular ejection fraction (LVEF) independently forecasted adverse outcomes post-DCB.

**Conclusion:**

In the context of ACS, diabetic patients remain uniquely vulnerable. Metabolic control and cardiac function—not anatomy alone—emerge as decisive prognostic factors after DCB therapy. Despite inherent challenges, DCB offers a targeted and promising revascularization approach when patient-specific risk modifiers are proactively addressed.

## Background

Acute coronary syndrome (ACS) continues to be a major global cause of cardiovascular mortality [1, 2]. The use of primary percutaneous coronary intervention (PCI) has markedly improved outcomes in patients suffering from acute myocardial infarction (AMI), particularly when administered without delay [3, 4]. Second-generation drug-eluting stents (DES) have become the standard of care in many settings, supported by extensive data validating their clinical safety and effectiveness [5, 6]. Comparative research has investigated the efficacy of drug-coated balloons (DCBs) versus conventional stenting, with some studies raising concerns about whether DCBs achieve similar clinical endpoints [7–9]. Nonetheless, emerging concerns over DES’s durability and long-term safety have led to growing interest in alternative therapies, such as DCBs. Originally introduced for the management of instent restenosis (ISR) [10, 11], DCBs have more recently been evaluated for their role in treating de novo coronary artery lesions. Several trials suggest that DCBs may offer comparable protection against major adverse cardiac events (MACE) when contrasted with DES [12, 13]. By delivering antiproliferative agents directly to the arterial wall without leaving behind a permanent scaffold, DCBs may minimize risks associated with implanted stents and may prove particularly advantageous in cases of small vessel disease [14].

Coronary revascularization in patients with diabetes mellitus (DM) is often more complex due to characteristic vascular changes, such as smaller vessel diameter and diffuse plaque buildup. These structural challenges can compromise stent efficacy and are linked to a higher incidence of restenosis after PCI. In light of these limitations, DCBs have gained attention as a potentially valuable treatment option for diabetic individuals presenting with ACS, as indicated by recent research [15]. While drug-eluting stents have significantly advanced in design and performance, diabetic patients continue to face increased risks of complications and mortality from coronary artery disease (CAD). This ongoing disparity highlights the urgent need for alternative, diabetes-specific revascularization strategies [16, 17].

Although the application of DCBs in percutaneous coronary interventions has gained increasing attention, there is still a notable scarcity of up-to-date research examining their role across the full range of ACS cases. Current evidence on the performance of DCBs in ACS settings remains sparse, particularly regarding safety and therapeutic outcomes. Diabetic patients, who often present with more challenging coronary disease, require distinct consideration, yet this subgroup is underexplored in existing studies. This study aims to critically evaluate the safety and efficacy of drug-coated balloon-only PCI in ACS patients, with a focused comparison between diabetic and non-diabetic cohorts, while unveiling the nuanced clinical outcomes within diabetic subpopulations to inform precision treatment strategies in this high-risk group, drawing on data from our institutional experience.

## Patients and methods

### Study design and participants

This retrospective cohort observational study was conducted at King Abdullah Medical City (KAMC), a tertiary care cardiac center. 212 patients presenting with ACS and managed with PCI using DCB angioplasty between January 2019 and December 2023 were included.

### Eligibility criteria

#### Inclusion criteria

This study enrolled ACS patients who underwent PCI of the culprit lesion using paclitaxel-DCBs, without the requirement for DES placement. All participants received optimal guideline-directed medical therapy and demonstrated adherence to prescribed medications.

#### Exclusion criteria

1. ACS patients managed medically without undergoing coronary angiography.

2. Patients who developed dissection following DCB application and required conversion to DES implantation.

### Study procedure

#### Interventional procedure

All patients received dual antiplatelet therapy before the intervention, which included aspirin (300 mg) combined with either ticagrelor (180 mg) or clopidogrel (600 mg), in addition to an intravenous infusion of unfractionated heparin at a dosage of 70–100 IU/kg. Lesion preparation during PCI followed standard protocols, with pre-dilatation mandatory for all cases. Semi-compliant balloons were used in approximately two-thirds of patients (67%), non-compliant balloons in 28%, and cutting or scoring balloons accounted for 5%. The decision to perform DCB angioplasty rather than DES implantation was based on specific angiographic and procedural considerations. DCB was applied only if pre-dilatation yielded residual stenosis ≤ 30% and no major coronary dissections beyond type C were present. Lesion preparation was performed using semi-compliant, non-compliant, or cutting/scoring balloons, depending on lesion characteristics, to optimize vessel morphology before DCB deployment. In cases where the angiographic outcome was suboptimal—such as significant dissection or high residual stenosis—a DES was implanted as a bailout. All participants received guideline-directed medical therapy and demonstrated adherence to prescribed medications, ensuring optimal procedural safety. These measures allowed DCB therapy to be reserved for patients in whom it was technically feasible and likely to provide effective outcomes, while DES was used for situations where DCB alone was insufficient.

#### Data collection

Patient information was comprehensively gathered across multiple clinical domains:

- **Baseline characteristics**: This included demographic details, initial clinical presentation, and traditional cardiovascular risk factors.
- **Echocardiographic assessment:** Measurements such as left ventricular ejection fraction (LVEF) and any notable valvular dysfunction were documented.
- **Angiographic evaluation:** Key parameters include the access site (femoral or radial artery), identification of the culprit vessel, and the types of balloons used—regular, noncompliant (NC), or cutting balloons. The size, length, and location (proximal, mid, or distal) of DCB inflations are meticulously recorded. Additionally, vessel caliber is classified as small if the diameter is less than 3 mm, and lesions shorter than 20 mm are categorized as short lesions. The number of affected coronary arteries and specifics of the interventional procedures were recorded.
- **In-hospital complications:** Events such as pulmonary edema, cardiogenic shock, cardiac arrest, and the necessity for mechanical ventilation were tracked during the hospital stay.
- **Primary clinical endpoint:** The main outcome measure was the incidence of MACE—a composite of cardiac death, myocardial infarction, stroke, and target lesion revascularization—assessed both during hospitalization and at six months post-intervention.

#### Statistical analysis

Data analysis was carried out using SPSS software, version 21.0 (SPSS Inc., Chicago, IL). Continuous variables are reported as means with standard deviations and were compared via the Student’s t-test. Categorical variables are expressed as percentages and analyzed using the chi-square test. Statistical significance was defined by a *p*-value below 0.05; values exceeding this threshold were considered non-significant. To identify independent predictors of major adverse cardiovascular events, a multivariate Cox regression analysis was performed.

## Results

**Fig. 1:**
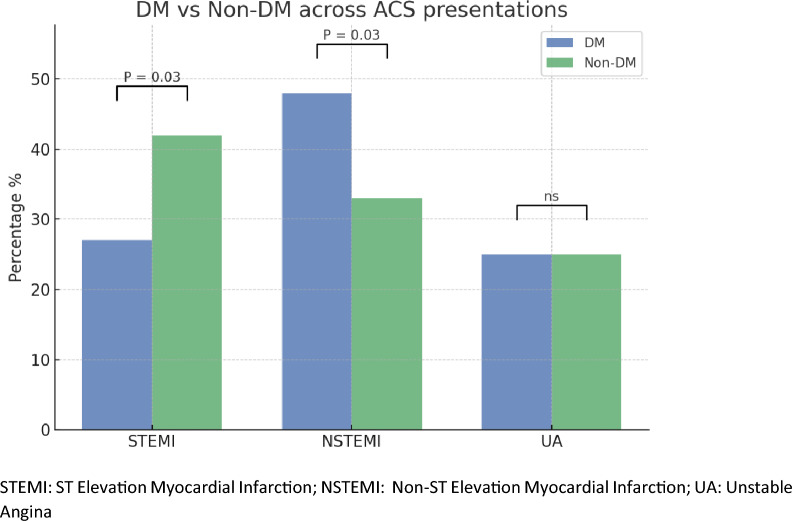
Comparison according to the acute coronary syndrome presentation. STEMI, ST elevation myocardial infarction; NSTEMI, Non-ST elevation myocardial infarction; UA, Unstable angina

Over a four-year enrollment period (2019–2023), a total of 212 individuals presenting with ACS were prospectively included in this analysis. Strikingly, more than half of the cohort (117 patients, 55%) had coexisting diabetes mellitus, underscoring the high burden of metabolic disease within this population. Among those with diabetes, 

+ The study population had an average age of 57.2 years (± 10.8). Baseline demographic and clinical variables are outlined in Table 1. Significant differences were identified between diabetic and non-diabetic patients regarding smoking prevalence, rates of hypertension, dyslipidemia, and prior percutaneous coronary intervention. Notably, non-ST-elevation myocardial infarction (NSTEMI) was more frequently observed among diabetic individuals (48% vs. 33%; *P* = 0.03), whereas ST-elevation myocardial infarction (STEMI) was more prevalent in the non-diabetic group (42% vs. 27%; *P* = 0.03), as shown in Fig. 1.

**Table 1:** Clinical profile of the ACS patients

Variable	Diabetic (*n* = 117)	Non-diabetic (*n* = 95)	*P*-value
Age	60 ± 11.6	57.9 ± 12	NS
Male	87 (74%)	80 (84%)	NS
BMI	28.92 ± 5.7	28.3 ± 5.7	NS
Smoking	33 (28%)	49 (52%)	**0.001**
Hypertension	96 (82%)	52 (55%)	**0.000**
Dyslipidemia	44 (38%)	20 (21%)	**0.010**
Obesity	44 (38%)	38 (40%)	NS
CVA	12 (10%)	9 (9%)	NS
Previous Revascularization	62 (53%)	28 (29%)	**0.002**

Statiscal comparison between the DM and non DM group showing that the Diabetic patients had significant lower prevelence of smoking and higher prevelence of HTN, DLP and previous revascurization (*P* value significant for all)

Data are presented as mean ± standard deviation, or n (%)

BMI, Body mass index; CVA, Cerbro vascualr accidents

### Revascularization-related characteristics

**Fig. 2:**
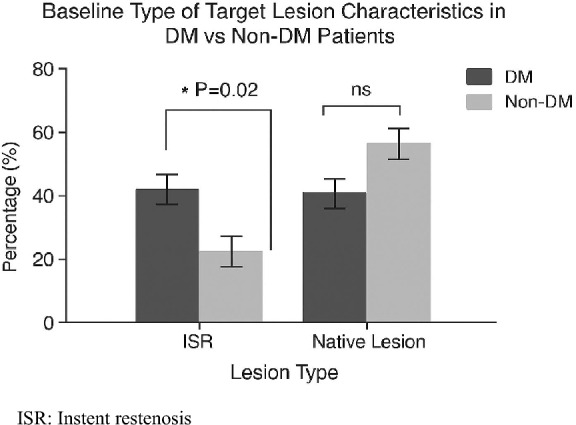
Baseline type of target lesion characteristics. ISR, Instent restenosis

Table 2 summarizes the key procedural parameters. While lesion characteristics and treated vessel locations showed no significant differences between groups, small vessel involvement was notably more prevalent among diabetic patients, prompting the more frequent use of smaller-diameter DCBs (75% vs. 55%; *P* = 0.01). Additionally, diabetic individuals were significantly more likely to undergo DCB angioplasty for in-stent restenosis compared to their non-diabetic counterparts (44% vs. 21%; *P* = 0.02), as illustrated in Fig. 2.

**Table 2:** Comparing baseline target lesion characteristics between groups

Variable	Diabetic	Non-diabetic	*P*-value
	(*n* = 117)	(*n* = 95)	
LAD	50 (43%)	32 (34%)	NS
Diagonal	11 (9%)	15 (16%)	NS
LCX	20 (17%)	13 (14%)	NS
OM	15 (13%)	9 (9%)	NS
RCA	21 (18%)	10 (11%)	NS
Small DCB diameter	88 (75%)	52 (55%)	0.01
Short DCB length	29 (25%)	31 (33%)	NS

Data are presented as *n* (%)

LAD, Left anterior descending artery; LCX, Left circumflex artery; OM, Obtuse marginal; RCA, Right coronary artery; DCB, Drug coated ballon

### Different groups and clinical outcomes

**Fig. 3:**
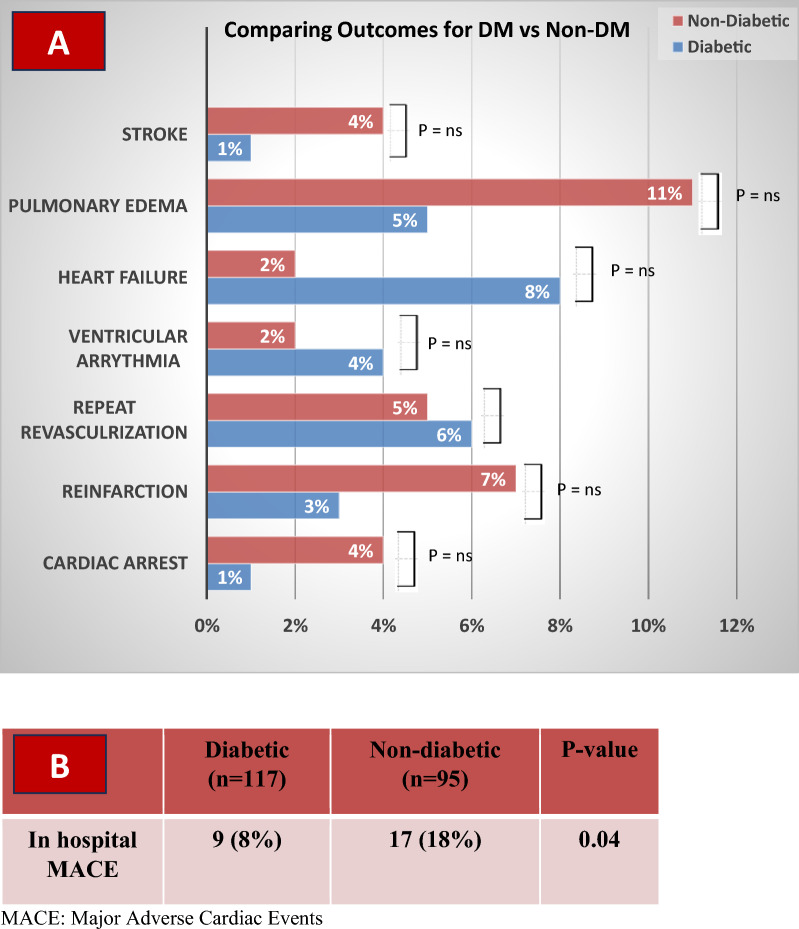
In-hospital major adverse cardiac events. MACE, Major adverse cardiac events

**Fig. 4:**
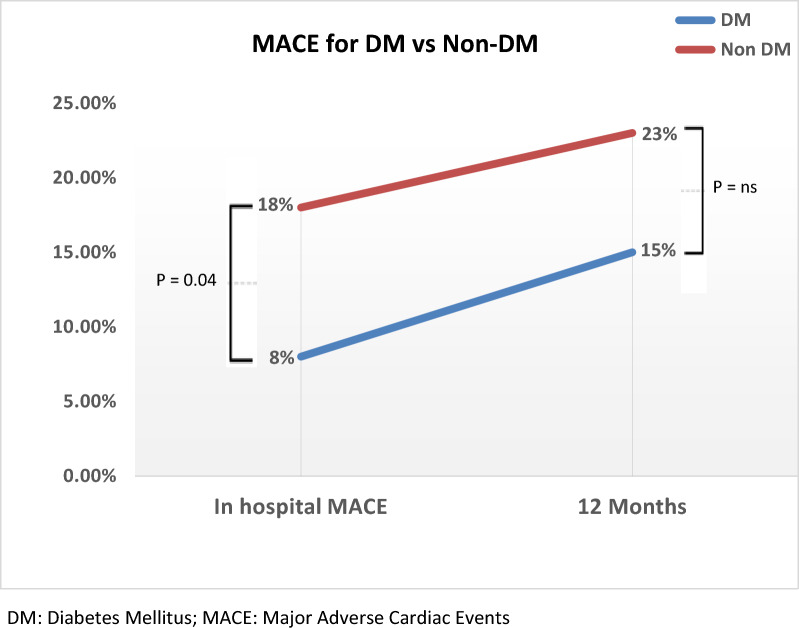
Comparing 1- and 12-month major adverse cardiac events. DM, Diabetes mellitus; MACE, Major adverse cardiac events

Analysis of in-hospital outcomes demonstrated that patients with diabetes who presented with ACS had a significantly lower rate of MACE compared to non-diabetic patients (*p* = 0.04) within the first month after the procedure. By twelve months, MACE rates between the two groups showed no meaningful statistical difference (refer to Figs. 3 and 4).

Despite comparable baseline demographics, clinical characteristics, ACS presentation, and coronary lesion distribution, insulin-treated diabetic (ITDM) patients with ACS undergoing DCB angioplasty experienced a disproportionately higher incidence of MACE compared to their non-insulin-treated diabetic (NITDM) counterparts (*P* = 0.04). This disparity was predominantly driven by a significantly increased need for repeat revascularization in the ITDM group (*P* = 0.04). Notably, the rates of reinfarction, cardiac arrest, and definite thrombosis remained similar between cohorts. Importantly, DCB demonstrated a reassuring safety profile across the spectrum of diabetic management, with consistently low thrombosis rates and no significant difference in fatal events, reinforcing its viability even in high-risk insulin-dependent populations (Table 3).

**Table 3:** Comparing clinical profile and outcomes of insulin- treated DM vs non-insulin-treated DM-ACS patients treated with DCB

Variable	Insulin-treated DM- ACS (*n* = 43) 37%	Non-insulin-treated DM-ACS (*n* = 74) 63%	*P*-value
Age	61 ± 11.6	59 ± 12.3	NS
Male	32 (74%)	55 (74%)	NS
BMI	28.92 ± 5.7	29.92 ± 4.5	NS
Smoking	12 (28%)	21 (28%)	NS
Hypertension	35 (82%)	61 (82%)	NS
Dyslipidemia	16 (37%)	28 (38%)	NS
STEMI	12 (28%)	20 (27%)	NS
LAD /Diagonal	23 (53%)	38 (51%)	NS
LCX/OM	14 (33%)	21 (28%)	NS
RCA	6 (14%)	15 (20%)	NS
Small DCB diameter	32 (74%)	56 (76%)	NS
Short DCB length	11 (26%)	18 (24%)	NS
In hospital MACE	3 (7%)	6 (8%)	NS
12-months MACE	9 (21%)	9 (12%)	0.04
Target lesion revascularization	5 (12%)	2 (3%)	0.04
Reinfarction	1 (2%)	2 (3%)	NS
Definite thrombosis	1 (2%)	0	NS
Cardiac arrest	1 (2%)	0	NS

Data are presented as *n* (%)

BMI, Body mass index; DCB, Drug coated balloon; LAD, Left anterior descending artery; LCX, Left circumflex artery; MACE, Major adverse cardiac events; OM, Obtuse marginal; RCA, Right coronary artery; STEMI, ST-Elevation myocardial infarction

**Fig. 5:**
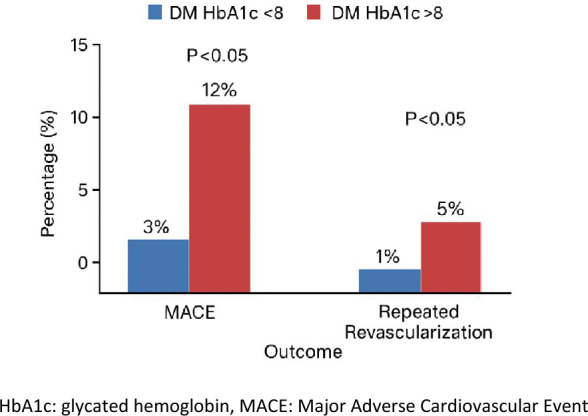
Comparing 12-month MACE and repeated revascularization rates by HbA1c level. HbA1c, Glycated hemoglobin, MACE, Major adverse cardiovascular event

In a stratified analysis of glycemic burden, among patients with diabetes mellitus undergoing DCB intervention for acute coronary syndrome, those presenting with glycated hemoglobin (HbA1c) levels above 8.0% demonstrated a markedly higher incidence of MACE and repeat revascularization at the twelve-month mark (*P* < 0.05). This trend suggests that inadequate glycemic control is critical in diminishing the durability of vascular healing and clinical benefit following stentless therapy (Fig. 5).

Furthermore, Multivariate analysis identified both elevated HbA1c levels and reduced LVEF at the procedure time as strong, independent predictors of twelve-month MACE (*P* = 0.005 and 0.002, respectively), underscoring their prognostic significance in determining adverse cardiovascular outcomes following PCI, as detailed in Table 4.

**Table 4:** Regression analysis for the independent predictors of twelve-month MACE

Variable	B	S.E	Wald	df	*P* value	Exp (B)	95% CI for Exp (B)
DM	7.84	5.73	1.87	1	0.170	2544.0	0.08–8.2 × 10⁷
Long stent > 20 mm	4.99	5.52	0.82	1	0.370	147.9	0.005–4.7 × 10⁶
HbA1c level > 8.0%	− 0.14	0.042	7.8	1	0.005**	1.15	1.04–1.28
Post PCI- LVEF	− 0.18	0.058	9.6	1	0.002**	0.835	0.743–0.937

Bold means that both HBA1c> 8 and low LVEF at procedural time are statistically significant varibles (paramaters) for 12 months MACE

DM, Diabètes mellites; HbA1c, Glycated hemoglobin; PCI, Percautenous coronary intervention; LVEF, Left ventricular éjection fraction

## Discussion

This study provides important insights into the performance of drug-coated balloon angioplasty in patients with ACS, particularly among those with diabetes mellitus. While diabetic patients exhibited anatomical and procedural differences, their overall long-term outcomes after DCB intervention were comparable to non-diabetic patients. Notably, diabetic patients experienced a lower rate of MACE during the first month post-procedure, a finding that initially appears counterintuitive. Several factors may help explain this early trend: diabetic patients were more likely to present with NSTEMI rather than STEMI, had higher rates of prior PCI, and showed strong adherence to guideline-directed medical therapy. Additionally, procedural elements such as careful lesion preparation and use of appropriately sized DCBs may have contributed to favorable early outcomes. Nonetheless, the influence of unmeasured confounding and the modest sample size cannot be excluded, and the early advantage was no longer apparent beyond the first month. This observation, therefore, should be interpreted cautiously and viewed as hypothesis-generating, highlighting the nuanced and sometimes unexpected behavior of diabetic patients undergoing DCB therapy. A further layer of complexity emerges when considering heterogeneity within the diabetic population. Patients requiring insulin or demonstrating poor glycemic control were at higher risk for adverse events, particularly repeat revascularization. These findings underscore the interplay between metabolic status and vascular response, emphasizing the importance of individualized strategies in high-risk diabetic patients treated with DCBs.

Diabetes continues to exert a significant adverse impact on cardiovascular outcomes despite advances in percutaneous interventions [18, 19]. Its unique vascular biology—including increased neointimal hyperplasia, persistent inflammation, endothelial dysfunction, platelet hyperactivity, and accumulation of advanced glycation end-products—contributes to higher restenosis rates and vascular injury [20–23]. Traditional DES, particularly with polymer coatings, may exacerbate these effects through prolonged vascular inflammation and stent underexpansion [24]. In this context, DCBs, by avoiding permanent implantation and delivering antiproliferative therapy directly to the lesion, may offer particular advantages in diabetic vessels [25, 26].

Although clinical data are limited, trials such as PEPCAD IV DM and EASTBOURNE DIABETES report favorable MACE outcomes with DCBs, despite slightly higher target lesion revascularization rates [27, 28]. Our findings align with these observations, suggesting that the absence of a stent scaffold may reduce early thrombo-inflammatory complications, while careful lesion preparation and optimal pharmacotherapy likely contribute to the early reduction in MACE seen in our diabetic cohort. Benefits of DCB therapy are also supported in broader ACS populations, including NSTEMI, where efficacy has been noted [29, 30].

Beyond procedural safety, DCBs provide homogeneous drug delivery while preserving vessel architecture, limiting chronic inflammation, and reducing neoatherosclerosis risk [14, 31, 32]. Their role is particularly relevant in diabetic patients with small vessel disease, diffuse calcified lesions, or in-stent restenosis. Our results reinforce these advantages, highlighting the therapeutic value of DCBs in anatomically complex and high-risk diabetic coronary lesions [33, 34].

A novel aspect of this study is the identification of insulin therapy as a predictor of adverse outcomes post-DCB angioplasty. Insulin-dependent patients experienced higher rates of cardiovascular events, consistent with prior DES data linking insulin use to greater atherosclerotic burden, impaired endothelial recovery, and compromised vessel function [35, 36]. In such high-risk patients, DCBs may provide a valuable stentless alternative, potentially reducing long-term inflammatory responses while supporting favorable vascular healing.

Glycemic control, as reflected by HbA1c, further influenced outcomes. Patients with HbA1c < 8.0% had better clinical trajectories, reinforcing the importance of metabolic optimization prior to intervention. Stratifying diabetic patients by insulin dependence and glycemic control may therefore improve prognostication and guide personalized treatment strategies [37].

Additionally, the combination of suboptimal glycemic control and reduced left ventricular ejection fraction (LVEF) emerged as a strong predictor of 12-month MACE. This dual-risk phenotype—metabolic dysfunction coupled with impaired cardiac function—substantially elevates cardiovascular risk, consistent with prior literature [38–40]. Integrating glycemic status and LVEF into pre-procedural evaluation could enhance risk stratification and inform tailored therapeutic approaches in high-risk diabetic patients.

In summary, our findings illuminate the intricate challenges of treating diabetic patients with ACS, particularly those with insulin dependence or poor glycemic control. Drug-coated balloon angioplasty appears to offer a safe and pragmatic strategy in this high-risk population, promoting vascular healing while circumventing complications linked to permanent stents. Importantly, these observations are exploratory and hypothesis-generating, highlighting potential patterns in early and longer-term outcomes rather than asserting definitive superiority. They underscore the need for larger, well-designed prospective studies to validate these trends, optimize patient selection, and refine personalized revascularization strategies for diabetic patients undergoing DCB therapy.

## Study limitations

This study offers important real-world insights but has several notable limitations. First, the retrospective and observational design may introduce unmeasured confounding, which limits causal inference and underscores the need for prospective evaluation. Second, no DES-treated control group was included. A retrospective comparison with DES could be heavily influenced by selection bias, as operator choice is affected by lesion complexity and patient characteristics. Our study was therefore deliberately focused on DCB-treated patients, providing focused insights into outcomes, particularly in diabetic ACS populations, where evidence remains limited. Third, only patients who underwent successful DCB PCI were included. Patients who either did not undergo PCI or required conversion to DES due to procedural complications, such as major dissection or high residual stenosis, were excluded. While this may underrepresent the highest-risk lesions, it allowed us to study a clearly defined cohort in which DCB therapy was technically feasible. Fourth, certain subgroup analyses, including insulin-treated versus non-insulin-treated diabetics and patients stratified by glycemic control, involved modest numbers. Although small, these subgroups provided clinically relevant observations and generated important hypotheses for future research. Fifth, the single-center setting and relatively small overall sample size may limit generalizability. Finally, the twelve-month follow-up provides only an initial perspective on outcomes; longer-term data are necessary to fully assess the durability and safety of DCB therapy. Overall, these limitations highlight the need for larger, multicenter, prospective studies including more complex patient populations and extended follow-up, to confirm and expand upon these findings and guide clinical decision-making.

## Conclusion

This study provides important insights into the use of DCB angioplasty for ACS, including among patients with diabetes mellitus—a population often considered high-risk due to complex lesions and increased incidence of in-stent restenosis. Outcomes in diabetic patients were generally comparable to non-diabetic individuals, highlighting the feasibility of DCB therapy in this subgroup. Insulin-dependent diabetics with suboptimal glycemic control exhibited a higher rate of MACE; however, low thrombosis and mortality rates reinforce the favorable safety profile of DCBs even in these higher-risk patients. Notably, reduced left ventricular ejection fraction and elevated HbA1c emerged as independent predictors of adverse events, emphasizing the need to integrate both cardiac function and metabolic status into clinical decision-making. Taken together, these findings should be viewed as hypothesis-generating, providing a foundation for future investigations. Larger, multicenter, prospective studies with longer follow-up are necessary to validate these observations, refine patient selection strategies, and optimize the long-term outcomes of DCB therapy.

## Data Availability

No datasets were generated or analysed during the current study.

## Abbreviations

ACS: Acute coronary syndrome
AMI: Acute myocardial infarction
CAD: Coronary artery disease
DCBs: Drug-coated balloons
DES: Drug-eluting stents
DM: Diabetes mellitus
HbA1c: Glycated hemoglobin
ISR: In-stent restenosis
ITDM: Insulin-treated diabetic
KAMC: King Abdullah Medical City
LVEF: Left ventricular ejection fraction
MACE: Major adverse cardiovascular events
NITDM: Non-insulin-treated diabetic
NSTEMI: Non-ST-elevation myocardial infarction
PCI: Percutaneous coronary intervention
STEMI: ST-ST-elevation myocardial infarction
